# Electronic Characterization of Au/DNA/ITO Metal-Semiconductor-Metal Diode and Its Application as a Radiation Sensor

**DOI:** 10.1371/journal.pone.0145423

**Published:** 2016-01-22

**Authors:** Hassan Maktuff Jaber Al-Ta’ii, Vengadesh Periasamy, Yusoff Mohd Amin

**Affiliations:** 1 Low Dimensional Materials Research Centre (LDMRC), Department of Physics, Faculty of Science, University of Malaya, 50603 Kuala Lumpur, Malaysia; 2 Department of Physics, Faculty of Science, University of Malaya, 50603 Kuala Lumpur, Malaysia; 3 Department of Physics, Faculty of Science, University of Al-Muthana, Al-Muthana 66001, Iraq; Drexel University, UNITED STATES

## Abstract

Deoxyribonucleic acid or DNA molecules expressed as double-stranded (DSS) negatively charged polymer plays a significant role in electronic states of metal/silicon semiconductor structures. Electrical parameters of an Au/DNA/ITO device prepared using self-assembly method was studied by using current–voltage (I-V) characteristic measurements under alpha bombardment at room temperature. The results were analyzed using conventional thermionic emission model, Cheung and Cheung’s method and Norde’s technique to estimate the barrier height, ideality factor, series resistance and Richardson constant of the Au/DNA/ITO structure. Besides demonstrating a strongly rectifying (diode) characteristic, it was also observed that orderly fluctuations occur in various electrical parameters of the Schottky structure. Increasing alpha radiation effectively influences the series resistance, while the barrier height, ideality factor and interface state density parameters respond linearly. Barrier height determined from I–V measurements were calculated at 0.7284 eV for non-radiated, increasing to about 0.7883 eV in 0.036 Gy showing an increase for all doses. We also demonstrate the hypersensitivity phenomena effect by studying the relationship between the series resistance for the three methods, the ideality factor and low-dose radiation. Based on the results, sensitive alpha particle detectors can be realized using Au/DNA/ITO Schottky junction sensor.

## Introduction

Deoxyribonucleic acid (DNA) has many advantages, mainly due to its vast accessibility, size manipulation capabilities and natural self-assembly properties [1]. However, the right genomic sequence, structure, environment and other properties are the pre-requisites necessary for generating optimized optical and electronic properties towards various stimuli [2, 3] leading to possible control of the properties [4]. For example, DNA molecules expressed as double-stranded (DSS) negatively charged phosphate group polymer and aromatic bases plays an important role in electronic states of metal/silicon semiconductor structures [1, 5]. Various other groups have also investigated and measured optimized DNA response towards magnetic field [6, 7], temperature [8], radiation [9, 10] and others [11].

Radiation has the ability to modify the flexibility of DNA. When irradiated using gamma rays and neutrons (non-ionizing radiation) the dynamics of DNA macromolecules [12] changes its configuration when involved in environmental interactions with other components of the living cells [13]. Electronic features of a Schottky diode is described by its barrier height, ideality factor and series resistance parameters. These features are in turn affected by factors like temperature [8, 14], interface [15], humidity [16, 17], and radiation [18, 19]. Whenever any radiation passes through a semiconductor device, different effects are observed depending on the range of energy of the particle (proton, alpha, neutron and both types of beta) and rays, such as gamma radiation [20]. These include defects such as vacancies, defect clusters, dislocation loops near the surface and adjustment of band gaps [21].

Electrical properties of DNA molecules can be understood by studying the electrical conduction mechanism. Three different mechanisms have been proposed; thermionic emission [22], tunneling and hopping [23, 24]. Recently, researchers studying the magnetic effect on DNA observed increase in the potential barrier between DNA and two gold (Au) wires as electrodes. Based on measurements of current-voltage (I-V) curve and Schottky’s rule, they suggested potential of the metal-DNA-metal (MDM) structures for utilization as sensitive magnetic sensors [6]. Güllü *et al* meanwhile used solution-processing method to fabricate a DNA organic-on-inorganic Schottky device. Based on the electrical properties of this device, effects of DNA interlayer on conventional metal/semiconductor [3] were also evaluated. In another work, Güllü *et al* in 2011 observed the effect of temperature on the current transport characteristics of Al/DNA/p-Si Schottky diodes in the range of 200–300 K [8]. Bellido *et al* analyzed I-V–temperature characteristics and observed current passing through a triangular DNA origami rely on the temperature when surrounded between two platinum electrodes [23]. Kulkarni *et al* recently discussed the feasibility of a DNA thin film as a radiation sensing material and considered it for sensing radiation based on reflected intensity variations of the exposed DNA thin film and biomolecules [9]. Thomas *et al* demonstrated that the reliability of the connection was improved when organic semiconductor buffer film was inserted between two metal electrodes and the DNA as gold/DNA/Pt. DNA could be destroyed by a direct metal evaporation process, which retards current production. According to Hassan *et al*, Ha *et al*, and Güllü *et al*, the electronic properties of DNA such as the I-V profiles are generally influenced by the contact, bulk (DNA channel) and intermolecular features under environmental conditions such as humidity effect and irradiation. It could also be assumed that the majority of response is initiated by the Schottky metal (Au) -semiconductor (DNA) contact. [19, 25, 26]. Conductivity sharply declines because of the long exposure in dry nitrogen or in vacuum. As such, it could be concluded that ion hydration shell and water molecules of the DNA plays a major part, with its conductivity evaluated at about 10^−6^–10^−5^ S·cm^-1^ per DNA molecule [27]. Okahata *et al* meanwhile measured the conductivity of the DNA placed on electrode comb to be 5.6 × 10^−5^ and 10^−9^ S cm^-1^ for current flow parallel and vertical to the aligned DNA strands, respectively [28]. The conductance was increased between two to five times when doping the metal ion onto the DNA crystals for some samples as such as with 4 mM of [Cu^2+^], 1.5 mM of [Ni^2+^], 1 mM of [Zn^2+^] and 0.6 mM of [Co^2+^] [29]. Recently, Dugasani *et al* observed that the resistivity tendency and Hall mobility declined when the ion concentration was raised while reaching the saturation concentration (C_s_) of each metal ion, thereby increasing the ion concentration. Also, they numerically discovered that the metal-DNA lattice behaved as a semiconductor with metal ions through the Hall parameters [30]. In this current work, we report the study of the current transport characteristics of Au/DNA/ITO Schottky diodes exposed to alpha radiation between 0–0.24 Gy. The current transport mechanism in these diodes was described using the features of forward I–V graphs. Additionally, we show the difference of the ideality factors, n, and the barrier heights, Φ at different exposure dose. The results obtained based on three methods (conventional, Cheung and Cheung’s and Norde methods) suggests the possibility of applying the Schottky junction as a sensitive sensor element to detect alpha particles.

## Experimental Section

### 2.1. Preparation of DNA solution

A simple preparation procedure of mushroom DNA extracted from colonies of fruiting bodies was used for Polymerase Chain Reaction (PCR) amplification. The DNA Kit was purchased from (OMEGA Bio-tek E.Z.NA.). The procedure starts with the collection of minute quantities of mycelium (0.1–1.0 g) from a colony of the fruiting body (Stipe) of a mushroom species using a sterilized tweezers. Standard procedures according to [31] were further employed to yield pure DNA samples prior to the PCR process. The DNA of all samples was amplified by PCR (PTC-100TM, MJ Research Inc., U.S.A.) using universal primers ITS1 forward (5′-TCC GTA GGTGAACCTGCGG-3′) and ITS4 reverse (5′-TCCTCCGCTT ATT GATATGC-3′). Amplification reactions were performed in a total volume of 50 μl containing 10x PCR buffer 4.0 μl, dNTP mix 2.5 μl, 2.5 μl of each primer, 1.0 μl of Taq polymerase (Cosmo, Korea), 4.0 μl of genomic template DNA, and 26.0 μl of sterilized distilled water. PCR amplification was carried out in 30 cycles at 94°C for 30 minutes and denatured at 50°C for 60 minutes followed by annealing at 72°C for an extension of 1 minute. Initial denaturing at 95°C was extended to 5 minutes and the final extension was at 72°C for 5 minutes [32, 33].

### 2.2. ITO cleaning procedures

ITO slides of width 1.1 mm purchased from KINTEC, Hong Kong had a layer thickness of 100 nm with a dimension of 2 cm x 2 cm and 377.0 Ω/sq and about 10^4^ S/cm of sheet resistance and conductivity, respectively. Thickness of the Au wire electrodes used was (0.50 ± 0.05) mm and purity 99.999% (Sigma Aldrich, USA). Prior to diode fabrication, the ITO substrates were cleaned thoroughly. In order to remove any oxides, the ITO slides were rinsed with soap in an ultrasonic bath for 10–15 minutes. The slides were washed using deionized water and dipped in acetone and iso-propyl alcohol (isopropanol, C_3_H_8_O) for 5 minutes each followed by final rinsing using deionized water. The chemicals were purchased from R&M chemicals (Essex, UK). Finally, the slides were dried using nitrogen gas to remove water and any other residues.

### 2.3. DNA film deposition

**Fig 1:**
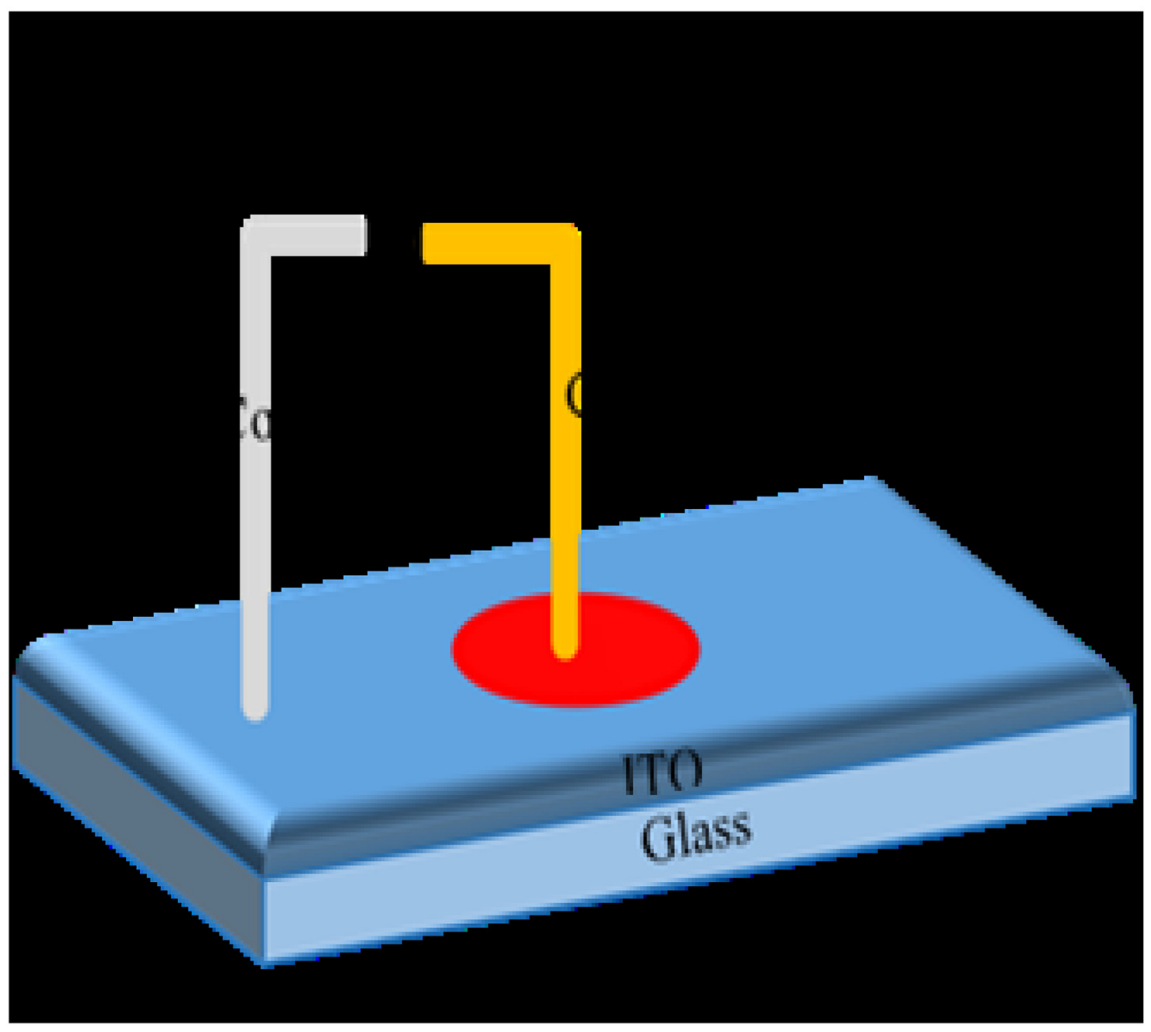
Cross-sectional view of Au/DNA/ITO Schottky diode.

DNA films were prepared by placing 10.0 μl of DNA solution of concentration of 1.80 ng/μl onto clean ITO glass substrates. The prepared DNA films were air-dried for 24 hours in a 1K clean room before irradiation. Sample irradiation by alpha particles was achieved using ^241^Am with an activity of 150 nCurie and t_1/2_ of 457 years for dose 0–0.24 Gy and the corresponding I-V profiles recorded. Fig 1 depicts the schematic diagram of the sensor fabricated for measuring the I-V characteristics (Keithley Electrometer, SMU236) and thickness of the DNA based on the Atomic Force Microscope (AFM) (Q-Scope Series, Ambios Technology, Germany) derived depth profiles of the DNA film. Width for non-radiated and 0.012 and 0.024 Gy of radiation were 150, 7.5 and 5.5 nm, respectively, demonstrating a reduction in DNA layer thickness with increasing dose.

## Results and Discussion

**Fig 2:**
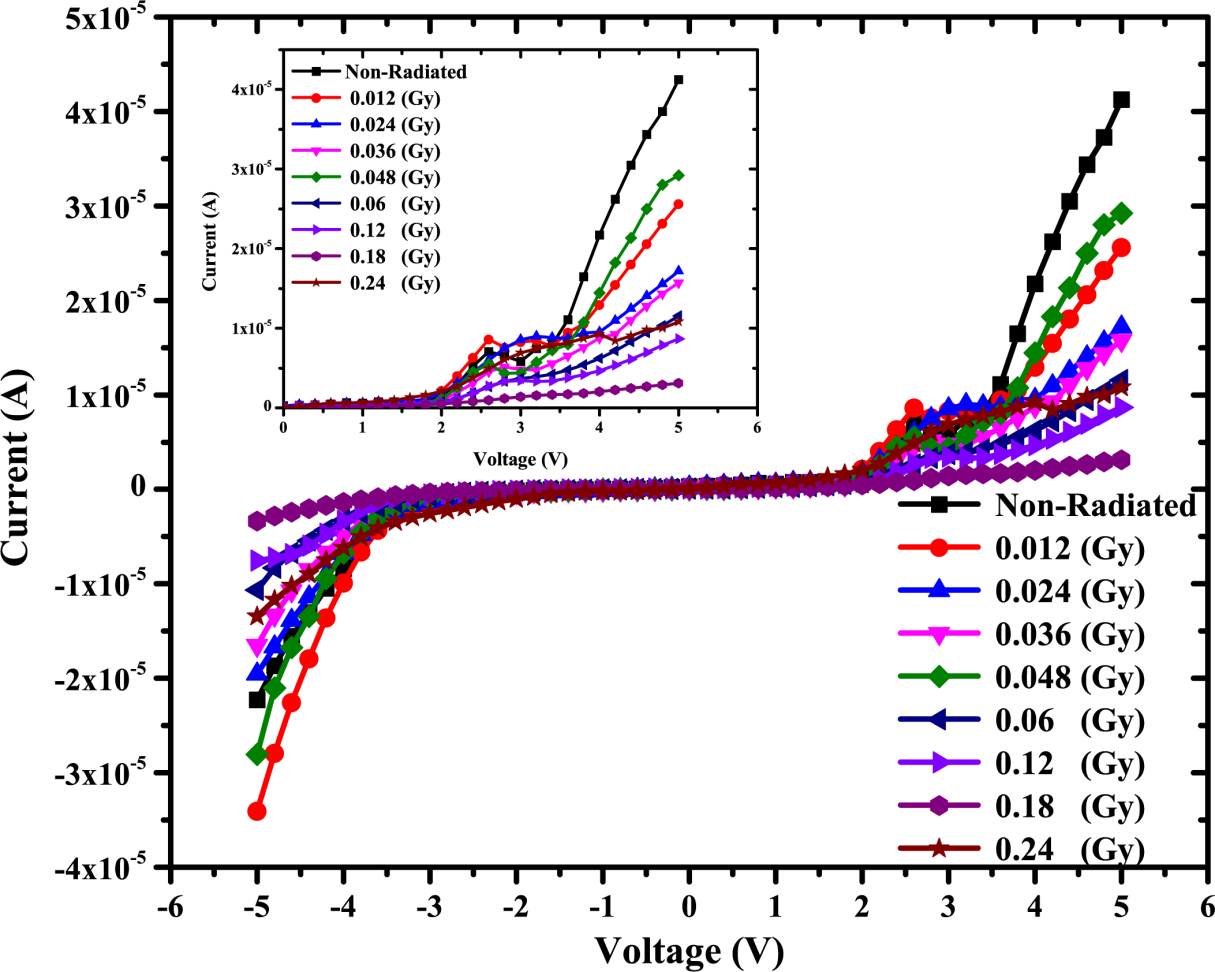
Current-voltage characteristics of Au/DNA/ITO Schottky structures.

I-V characteristics of the Au/DNA/ITO (MS) Schottky structure at 300K (room temperature) are shown in Fig 2, described by the following relations [22, 34–38];

$$I=I_{ο}\exp\left(\frac{qV}{nKT}\right)\left[1-\exp\left(\frac{-qV}{KT}\right)\right]$$ (1)

and

$$I_{ο}=AA^{*}T^{2}\left(\frac{-q\Phi }{KT}\right)$$ (2)

where I_o_ is the saturation density, V is the definite forward-bias voltage, A is the effective diode area, K is the Boltzmann constant, T is the absolute temperature, A* is the Richardson constant for Au (A* = 134.95 Acm^-2^K^-2^) [39].

**Fig 3:**
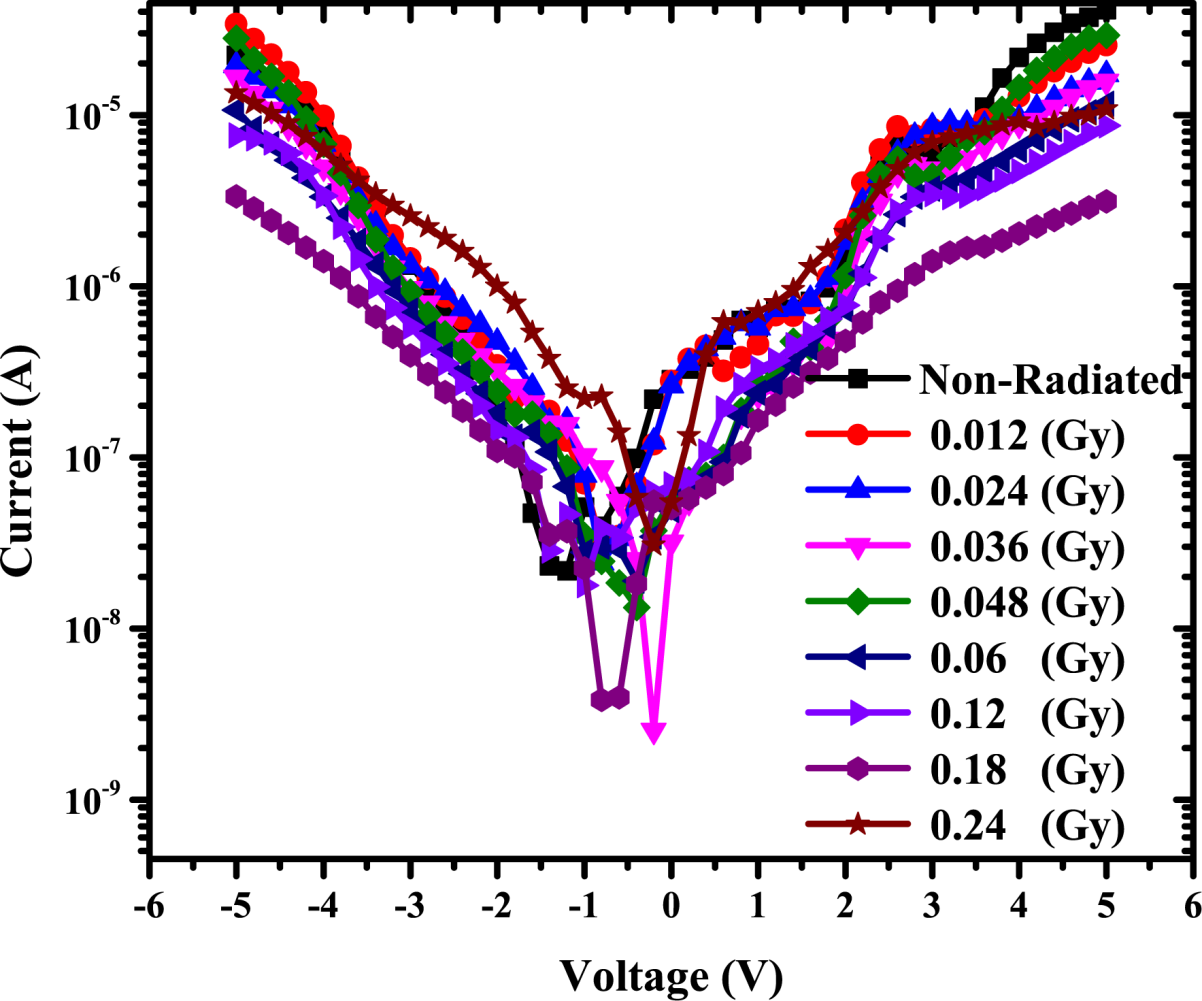
Forward and reverse bias I-V characteristics of the Au/DNA/ITO structures at room temperature.

The barrier height was calculated using Eq (2). The value of reverse saturation current is obtained from forward bias semi log (I)-V for all cases. Value of ideality factor, n was calculated from the slope of the linear region of the forward bias ln(I)-V plot in Fig 3 by using the following relation [40];

$$n=\frac{q}{KT}\left(\frac{dV}{d\;\ln\;I}\right)$$ (3)

The values of the ideality factors are presented in Table 1. For an ideal Schottky barrier diode, n equals to 1. However, n has usually a value greater than unity. This value indicates that the effect of the series resistance in the linear region is important. Higher values of n may also be due to non-homogeneous thickness of organic film or light effect [36, 40]. The connection characteristics of DNA based diode rely on the thickness [41] and temperature [36, 42]. From Eq (2), the barrier height, Φ can be derived and shown by;

$$\Phi =\frac{KT}{q}\ln\left(\frac{AA^{*}T^{2}}{I_{ο}}\right)$$ (4)

**Table 1:** Values of ideality factor, barrier height and series resistance measured. Uncertainties are shown for the Cheung and Cheung’s Method obtained through curve fitting while no such values are shown for the other two models since it was calculated from the equations.

Dose (Gy)	Conventional Method			Cheung and Cheung’s Model				Norde Method	
			dV/dI	dV/dlnI		H(I)-I		F(V)-V	
	n	Ф (eV)	R_s_(MΩ)	n	R_s_(MΩ)	Ф (eV)	R_s_(MΩ)	Ф (eV)	R_s_(MΩ)
Non-Radiated	1.35732	0.7284	0.120821	8.8803	0.0064±0.000157	0.6679	0.011± 0.04468	0.7230	0.04244
0.012	1.37498	0.7600	0.171260	8.1081	0.0099±0.00113	0.685	0.028±0.00173	0.7201	0.04742
0.024	1.17205	0.7371	0.219648	7.3359	0.0120±0.000242	0.6653	0.043±0.002037	0.6732	0.06339
0.036	1.48465	0.7879	0.316129	6.9498	0.0180±0.00154	0.6895	0.096±0.00196	0.6568	0.11504
0.048	2.2488	0.7426	0.1711	3.4486	0.0154±0.001388	0.7172	0.066±0.00538	0.7188	0.04480
0.06	1.34893	0.7798	0.4329	2.2890	0.0392±0.002599	0.6926	0.078±0.0111	0.6962	0.11991
0.12	1.26426	0.7491	0.5768	1.6162	0.0534±0.003274	0.6819	0.148±0.0154	0.6963	0.12021
0.18	2.4033	0.7407	1.6	1.3019	0.128±0.005966	0.7007	1.012±0.0382	0.6744	0.24370
0.24	2.35403	0.7670	0.4333	1.4324	0.0356±0.001425	0.6794	0.24±0.0143	0.7142	0.05281

n = Ideality factor, Ф (eV) = Barrier height,Rs = Series resistance.

From Fig 3, the deep valley demonstrates non-zero current at 0 V for the samples with an offset-shift in the negative direction up to 0.2 and 0.8 V for the 0.036 and 0.18 Gy, respectively. It might be attributed to the alpha particle charge or dose (storage, trapping, and de-trapping of the charge carriers) agreeing with Dugasani *et al* [30] and with Reddy *et al* in terms of the creation of reverse molecule charge interaction between DNA and the metal [43].

**Fig 4:**
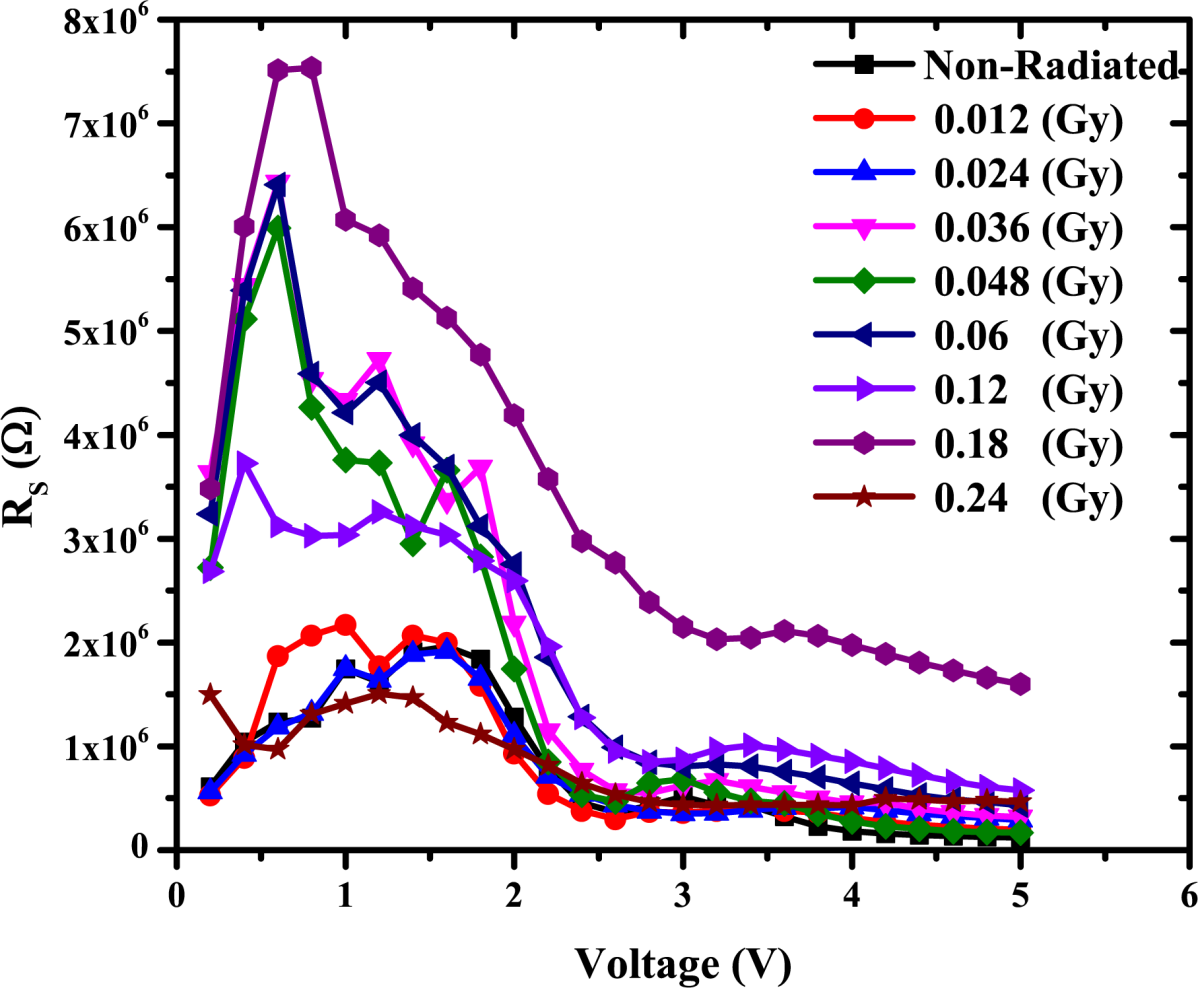
The relation between the series resistance versus voltage as calculated using the conventional method.

Using Eqs (3) and (4), the values of barrier height (Φ_b_) and the ideality factor (n) of the gold/DNA/ITO Schottky diode were obtained. The barrier height describes the potential barrier at the interface of the DNA and gold wire. Φ_b_ will limit the flow of charge carriers in the device. The highest values of Φ_b_ were related to non-uniform junctions at the interface. In addition, Φ_b_ and n rely on the interfacial layer thickness, the interface state density and series resistance [44, 45]. The values of series resistance is calculated from the junction resistance from Rs=∂V∂I from the I-V properties of the diode. The resistance R_S_ versus voltage of the surface-type (ITO/DNA/Au) Schottky diode is demonstrated in Fig 4. From the figure, it can be seen that at low voltages (≤2.0V), R_S_ values become the lowest for all the doses except for 0.18, 0.6, 0.048 and 0.036 Gy. However, above 2.0 V, the R_S_ values become insignificant for the latter sample. The highest R_S_ value occurs in the 0.18 Gy sample followed by 0.12 and 0.06 Gy.

The second method used in this study is the Cheung and Cheung’s model, which also considers the three parameters; series resistance, the barrier height and the ideality factor. Cheung and Cheung’s formula can be written as follows;

$$\frac{dV}{d\;\ln\;I}={IR}_{s}+n\frac{KT}{q}$$ (5)

$$H(I)=V-\left(\frac{KT}{q}\right)\ln\left(\frac{I}{{AA}^{*}T^{2}}\right)$$ (6)

therefore

$$H(I)={IR}_{s}+n\Phi_{b}$$ (7)

**Fig 5:**
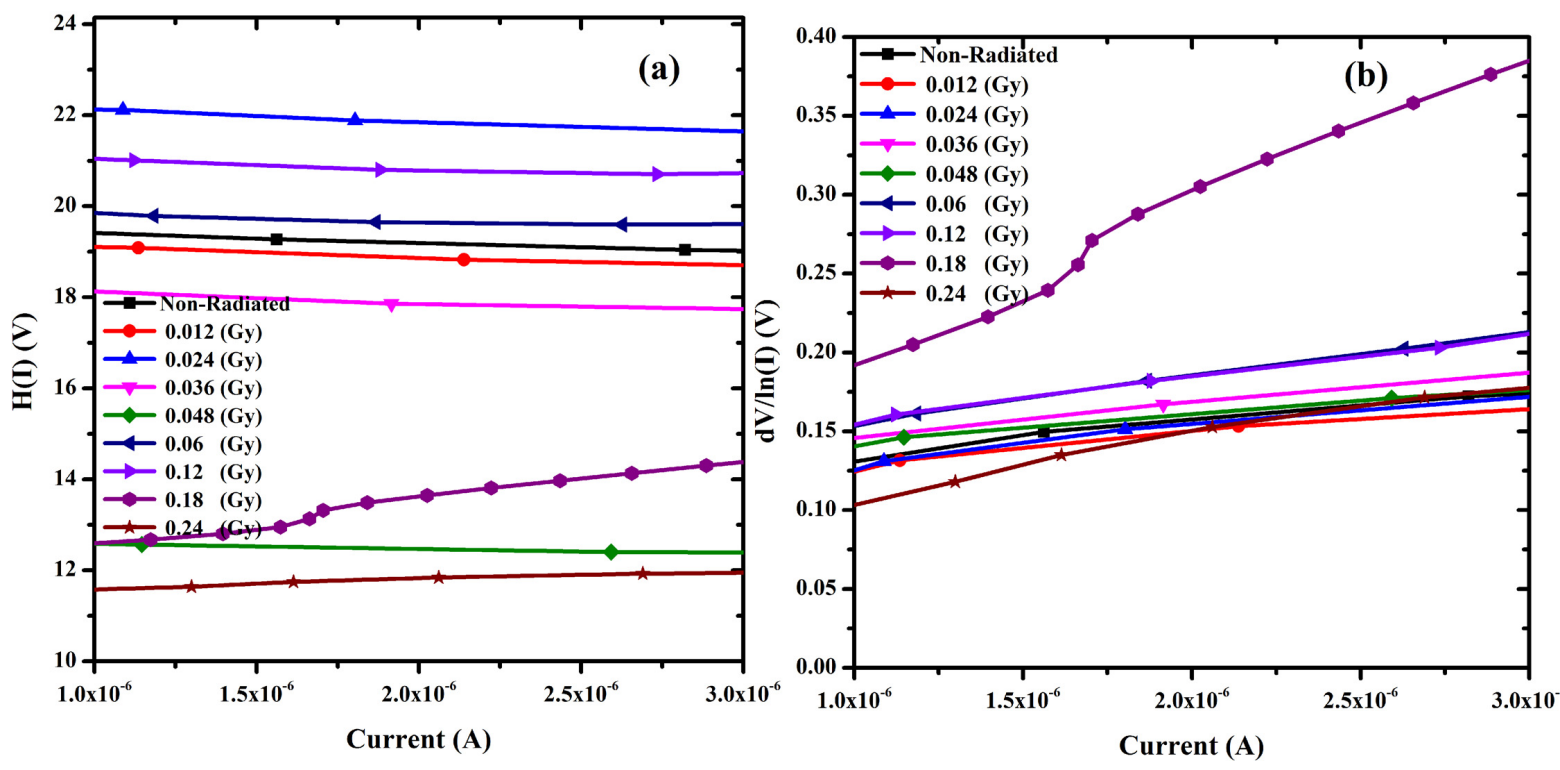
(a) H(I) vs I and (b) dV/d(ln I) vs I characterization of Au/DNA/ITO Schottky junction diode.

Fig 5(A) and 5(B) shows the experimental H(I) versus I and dV/d(lnI) versus I plots respectively at room temperature. The former graph demonstrates a straight line intercepting at y-axis equaling to nΦ. Φ was obtained by substituting the n value from Eq 5 and the data of the downward curvature region in the forward bias I-V using Eq 7. The slope of this plot also limits R_S_, which can be utilized to check the accuracy of Cheung and Cheung’s method. From H(I) versus I, the Φ and R_S_ values were measured and presented in Table 1. Eq 5 gives a straight line for the data of the downward curvature region in the forward bias I-V properties. Fig 5(B) shows the plot of dV/d(lnI) versus I, from which the values of n and R_S_ were calculated (Table 1). As can be seen from the table, the values of R_S_ obtained from dV/d(lnI) versus I and H(I) versus I plots are in near agreement with each other.

Radiation dose however does play an important role in changing resistance values, thus the resistance increases gradually at low doses, which therefore makes the DNA seek self-protection. The real barrier height, (Φ_b_) was taken from the low-voltage part of forward I-V graph while the series resistance from the straight-line region of Fig 5(A). Then the values of barrier height and the series resistance were obtained from Eq (7). All values from the conventional and the Cheung-Cheung’s model are presented in Table 1 showing a general fluctuating trend with radiation dose.

Norde method meanwhile can be used as an alternative method to calculate values of the series resistance and barrier height [46]. The following function has been defined in modified Norde's method;

$$F(V)=\frac{V}{\gamma }-\frac{KT}{q}\ln\left(\frac{I}{{AA}^{*}T^{2}}\right)$$ (8)

**Fig 6:**
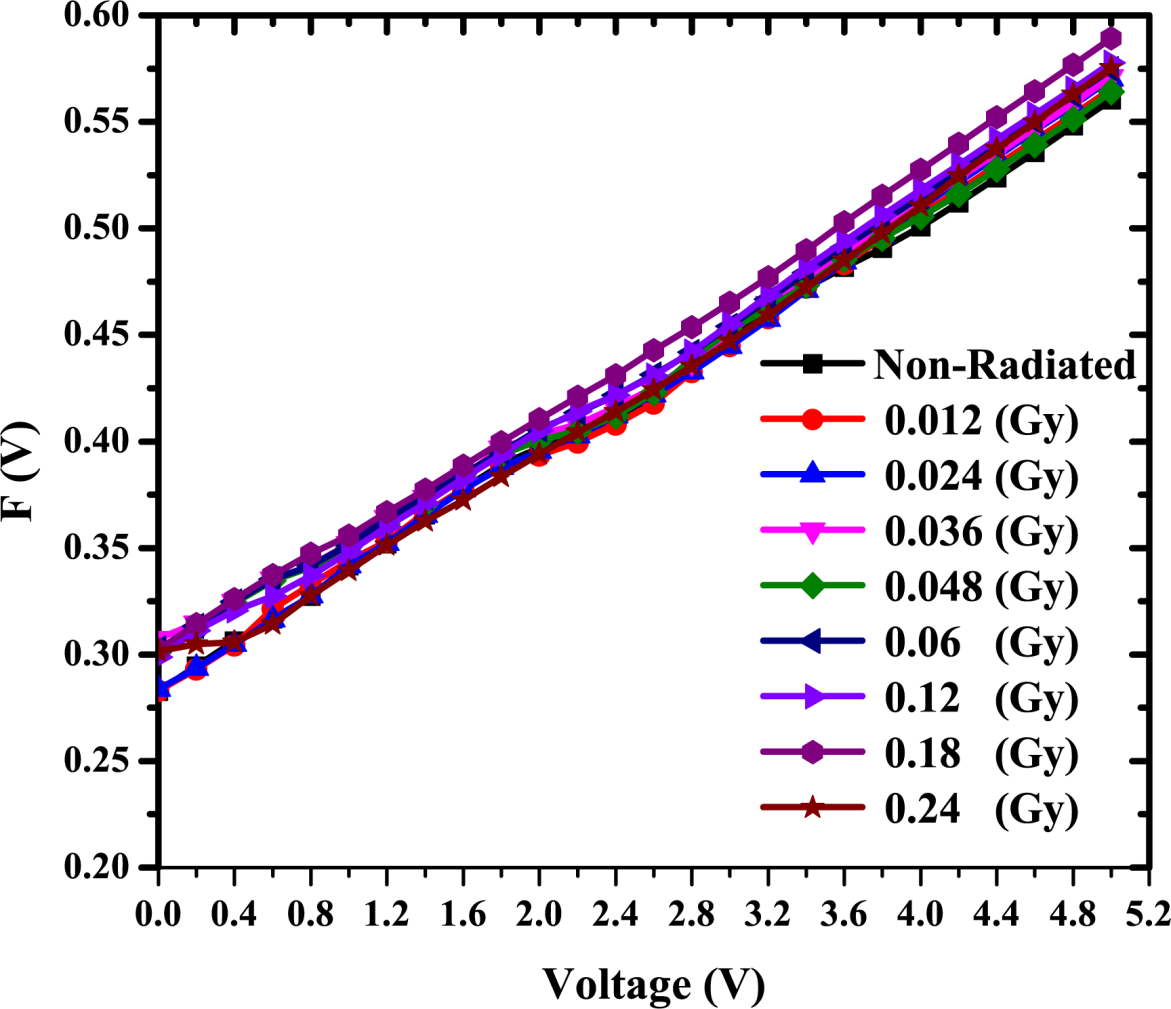
F (V) versus applied voltage of the Au/DNA/ITO device Schottky diode.

The effective Schottky barrier height is therefore given by;

$$\Phi =F(V_{\min})+\frac{V_{\min}}{\gamma }-\frac{KT}{q}$$ (9)

and

$$R_{s}=\frac{(\gamma -n)KT}{{qI}_{ο}}$$ (10)

where F(V_min_) is the minimum point in the F(V) versus V curve, V_min_ and I_o_ are the corresponding voltage and current respectively. A plot of F(V) versus V for Au/DNA/ITO Schottky structure at room temperature is as shown in Fig 6. From the plot F(V) versus V, the values of Φ and R_S_ of the structure were determined and tabled (Table 1).

**Fig 7:**
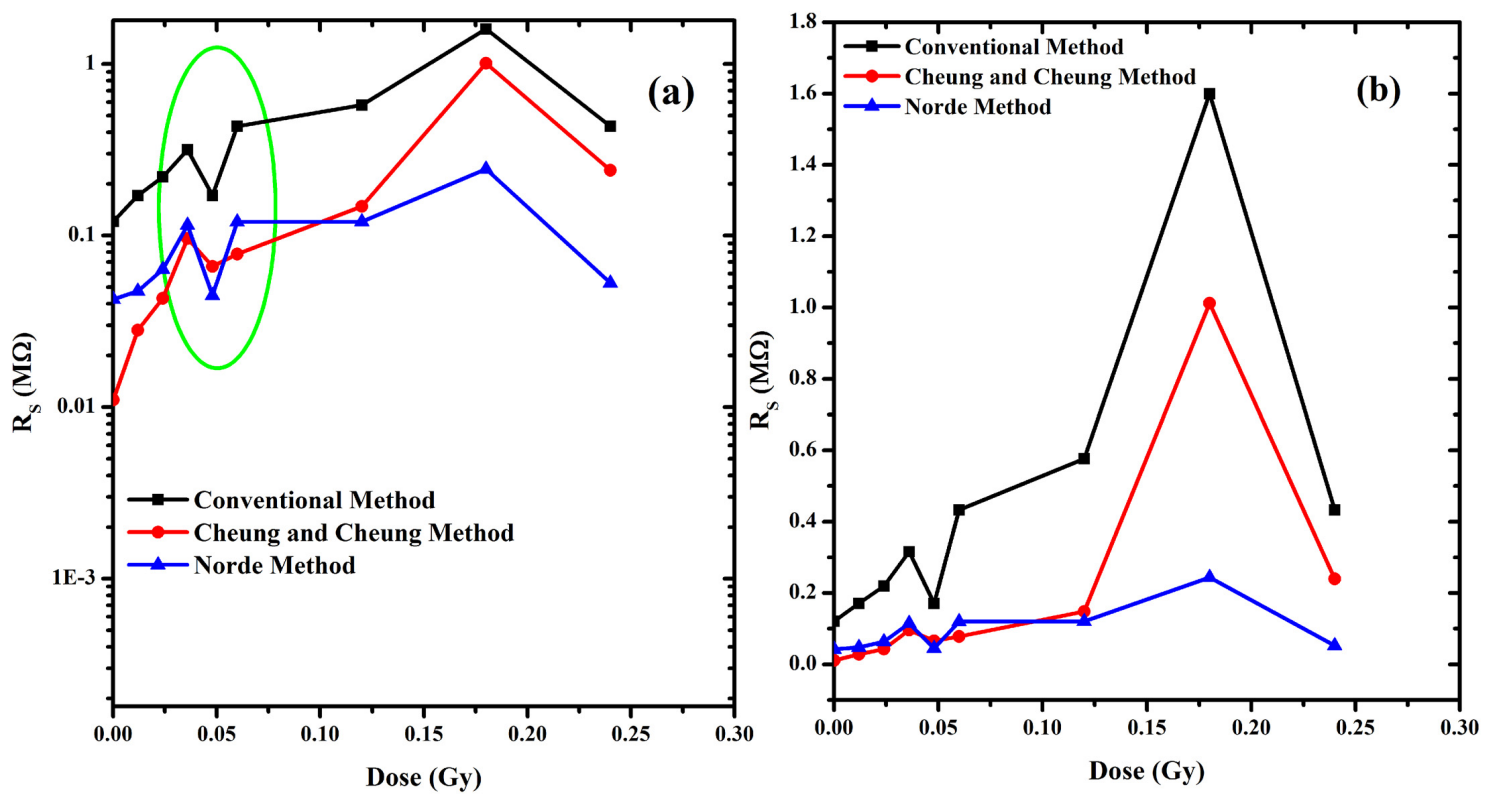
(a) Relation between series resistance and dose. (b) Relation between log series resistance and dose.

**Fig 8:**
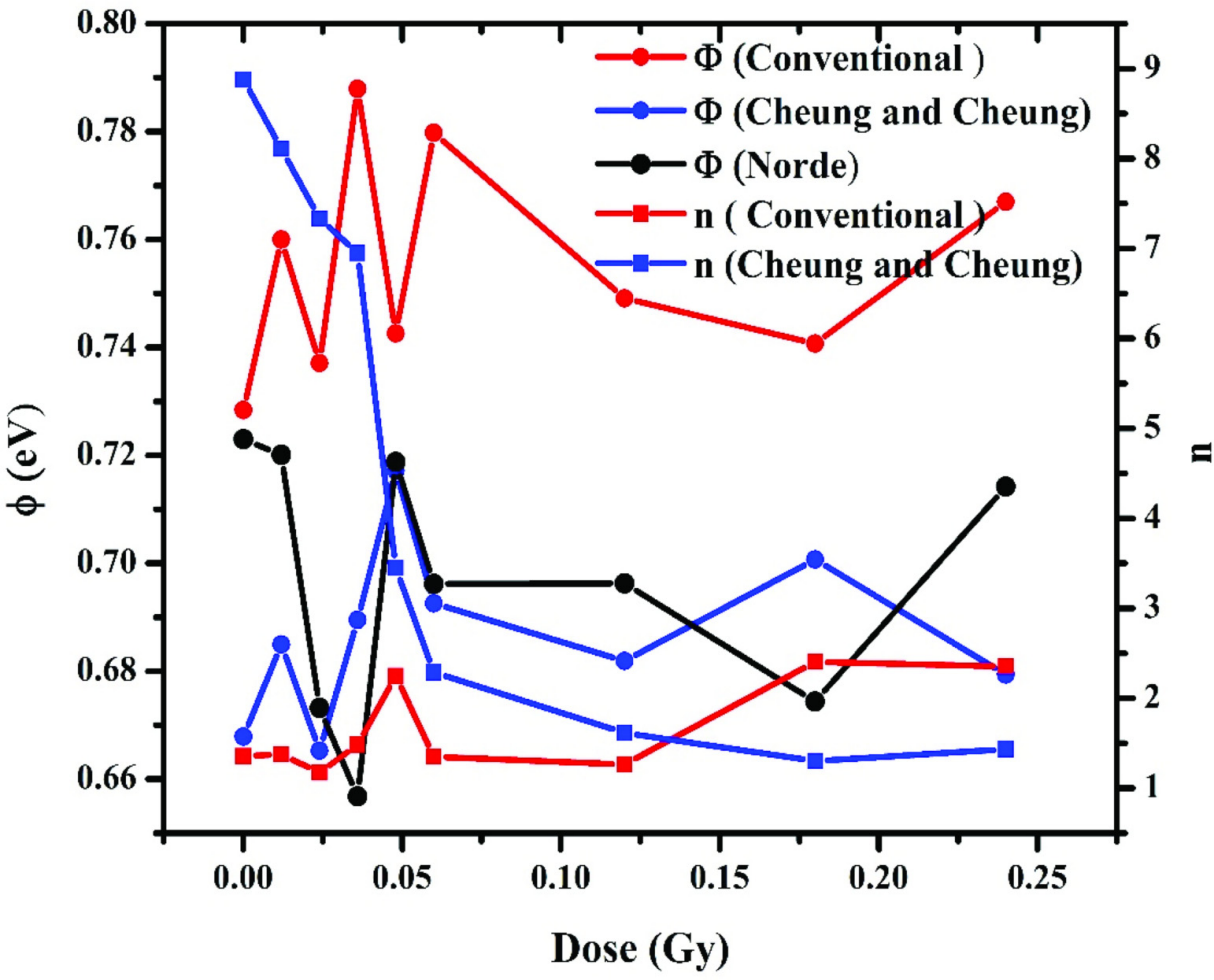
The relation between ideality factor and barrier height with radiation time.

Plots of Φ, n and R_S_ with the radiation time shown in Figs 7(A) and 7(B) and 8 indicate the hypersensitivity phenomena of the DNA at low radiation dose. 0.4480 Gy.

The values were found to be the lowest using Cheung and Cheung’s model compared to the other two methods with a general increase with the radiation dose. The values however fluctuate for barrier height in the case of Cheung and Cheung’s model. Values of barrier height using Conventional method were closer to Norde’s method, where the former have the lowest values.

**Fig 9:**
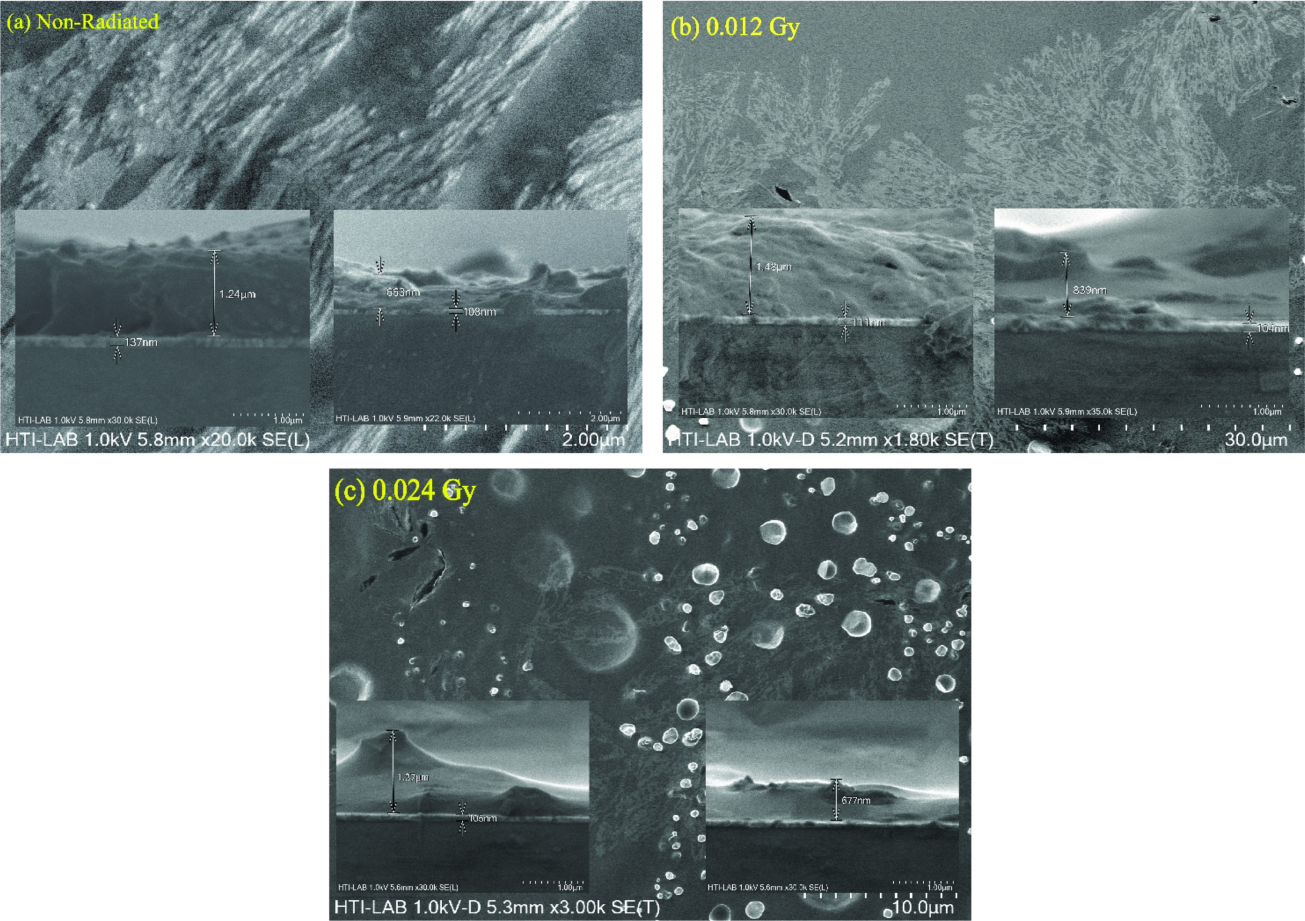
FESEM images of radiated and non-radiated samples.

From Fig 7(B), series resistance was found to increase with increasing radiation dose except in 0.05 Gy, probably due to the increasing number of alpha particle tracks (Fig 9). The thickness of the DNA film is as shown in Fig 9. Observations indicate general value of 1.24 μm with occasional dips of around 663 nm for the non-radiated sample. This increases to about 1.48 μm and 839 nm for the 0.012 Gy radiated sample. Further alpha radiation of 0.024 Gy reduces these values to 1.37 μm and 677 nm. This could be attributed to the alpha particles tracks, the hypersensitivity phenomena and due to the drop casting method used in this study.

At low doses, the R_S_ drop dramatically demonstrating the hypersensitivity phenomena of DNA Fig 8, which acts to protect itself. With further increase in time-dependent dosage, this phenomenon is similar to the relation between survival curve and dose [46–48]. By increasing the irradiation dose and conducting similar experiments for periods of 0, 2 and 4 min and the width for non-radiated and 0.012 and 0.024 Gy of irradiation dose are 150, 7.5 and 5.5 nm, respectively [10]. It was very clear in the conventional method that the Schottky barrier height on the other hand had an inverse proportionality relationship with the ideality factor as observed in Fig 8.

**Fig 10:**
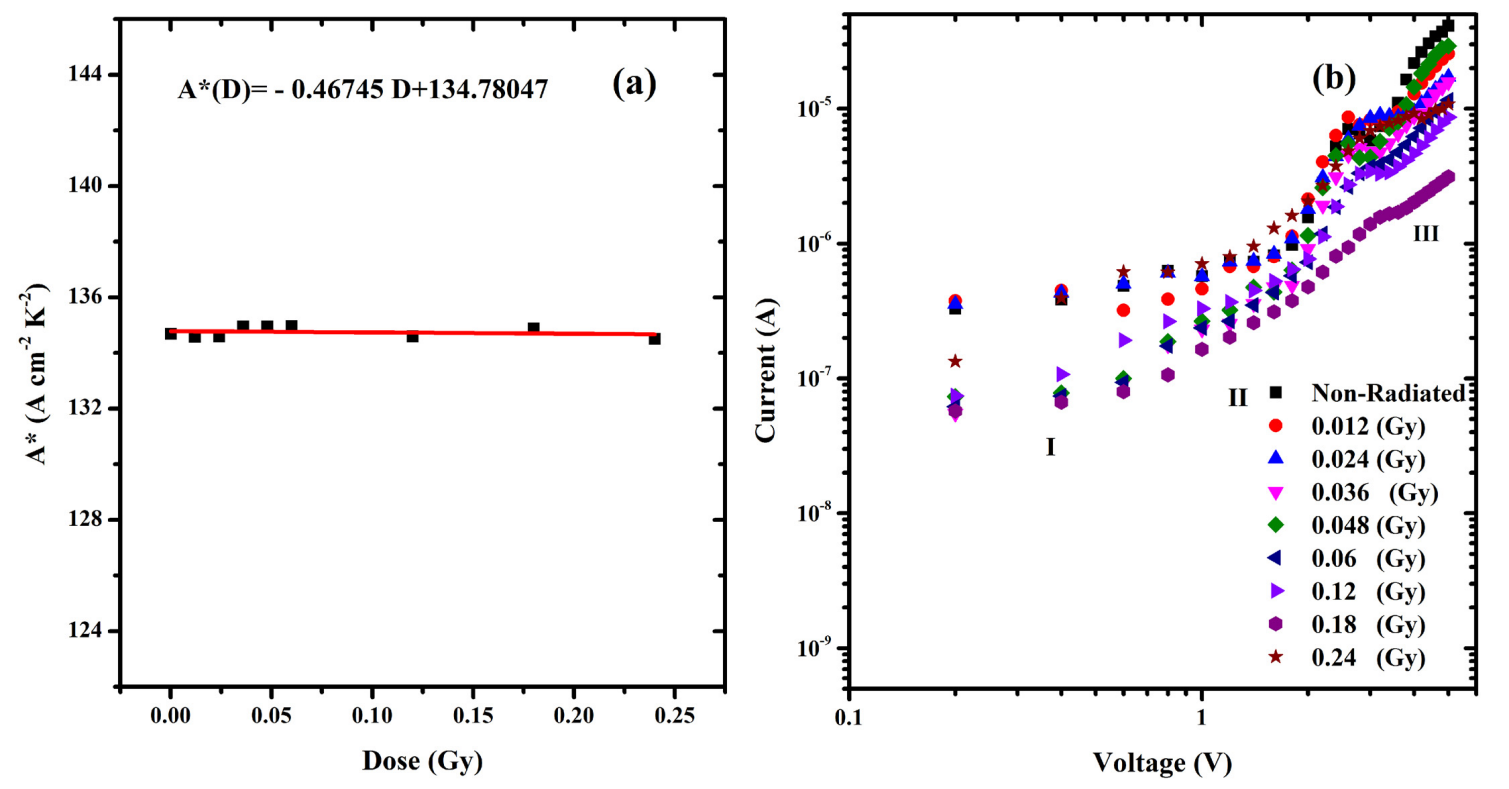
(a) Irradiation time dependent Richardson constant for the MDM structure and (b) double logarithmic plots of the ITO/DNA/Au junctions.

Richardson constant was determined from the I-V curve, and it rapid changes with dosage show Fig 10(A). The ionizing radiation process leads to energy sedimentation in the metal, denoted as thermal heat which changes the material characterization [20]. The work function of the metal/semiconductor junction changes, which affords sufficient energy for the charge carriers to get over the binding potential. Rising number of alpha particles tracks also leads to creation of higher number of holes, thereby increasing the effective mass. Since only a lower number of carriers are able to break through the potential barrier now, this would translate into a decrease in the current.

Due to the excitation of the material by ionizing radiation, such as by alpha particles, initially outputs an enormous number of excited atoms along its path, thereby increasing the number of electrons. However, continuous irradiation results in a decrease in the number of electrons as a result of collisions between the metal-DNA-metal (MDM) electrodes and the rise in resistance due to the number of traps in DNA preventing internal charge movement and hence increase the electrical resistance [49, 50]. The number of electrons also decreases through the collision between the metal-semiconductor-metal (MSM) electrodes. This results in the growth of the barrier heights as in Table 2, followed by a decline in the current. Fig 10(B) exhibits dual logarithmic plot of forward bias I-V features of the ITO/DNA/Au junction. The log (I)-log (V) graphs clearly shows the power law performance of the I-V curve.

**Table 2:** Barrier height and Richardson constant (A^*^) against irradiation time in ITO/DNA/Au (ɸ) structures.

Dose (Gy)	Richardson constant	Barrier Height
	A* (A.cm^-2^.K^-2^)	Ф (eV)
Non -Radiation	134.69052	0.7284
0.012	134.57489	0.7603
0.024	134.59185	0.7374
0.036	134.94212	0.7883
0.048	134.94788	0.7426
0.06	134.95653	0.7798
0.12	134.5969	0.7491
0.18	134.88262	0.7407
0.24	134.50431	0.7670

A* (A.cm^-2^.K^-2^) = Richardson constant, Ф (eV) = Barrier Height.

Space-charge-limited current (SCLC) effects the diode and its charge transport can be displayed through the I = V^m^ rule where m is the slope of each part, which harmonize to ohmic and SCLC. The m values shown in Table 3 portray three linear parts of the log (I) -log (V) plot of the forward bias I-V characterization. Part (I) shows an ohmic part, while the second part (II) demonstrates the presence of the SCLC mechanism controlled by the traps. The second part of this graph has slope values of between 1.9–4.8 up to a transition voltage of about ≈ 2.7 V, similar to the SCLC with the exponential distribution of traps in the band gap of the organic material The third part of the double logarithmic forward bias curve has slope values of between 1.7–4.19 except for the sample with 0.24 Gy of radiation. This part shows that at higher voltages, the slope of the curve declines since the device approaches the trap filled limit.

**Table 3:** Values of (m) for parts (I), (II) and (III) of the power law ITO/DNA/Au.

Dose (Gy)	ITO/DNA/Au Junction		
	(slope gradient,m)		
	Region (I)	Region (II)	Region (III)
Non -Radiation	0.45039	4.8048	4.19712
0.012	0.04507	3.552	2.8509
0.024	0.33099	3.63131	2.16649
0.036	0.88686	3.79171	2.69853
0.048	0.78422	3.26422	3.99268
0.06	0.8245	2.6816	2.37995
0.12	0.95396	2.49081	2.73198
0.18	0.41336	1.92962	1.73113
0.24	1.41163	2.16874	0.84589

The photoluminescence (PL) spectra of irradiated DNA were obtained. A red shift occurs at a range of 610 to 750 nm higher than the green wavelength (500–570 nm) due to the irradiation effect. The intensity of these peaks increases with an increase in the radiation dose. Luminescence phenomena involve subsequent light emission and energy absorption. Phosphors are luminescent materials that produce light after excitation by radiation, thin films or micro-crystalline powders, which usually produce visible color emission. This confirms that the purified DNA has a five-component linear triple complex involves of green, orange and red dots in the prepared. According to Tikhomirov *et al*, the complex exhibits pure red luminescence and is substantially devoid of the green and orange luminescence that was present before hybridization, and the linear ternary complex after cleavage by DNase, displaying the reversibility of complexation [51]. It is known that the emission spectrum of the nucleic acid bases (adenine, guanine, cytosine, thymine, uracil) is in the range of 400–600 nm, which indicates that the major contribution to the DNA PL spectrum are caused by these bases [48]. The homogeneous and specific bindings to DNA against intercalation between base-pairs and binding on phosphate backbones lead to the enhancing of the intensity of the PL [52].

**Fig 11:**
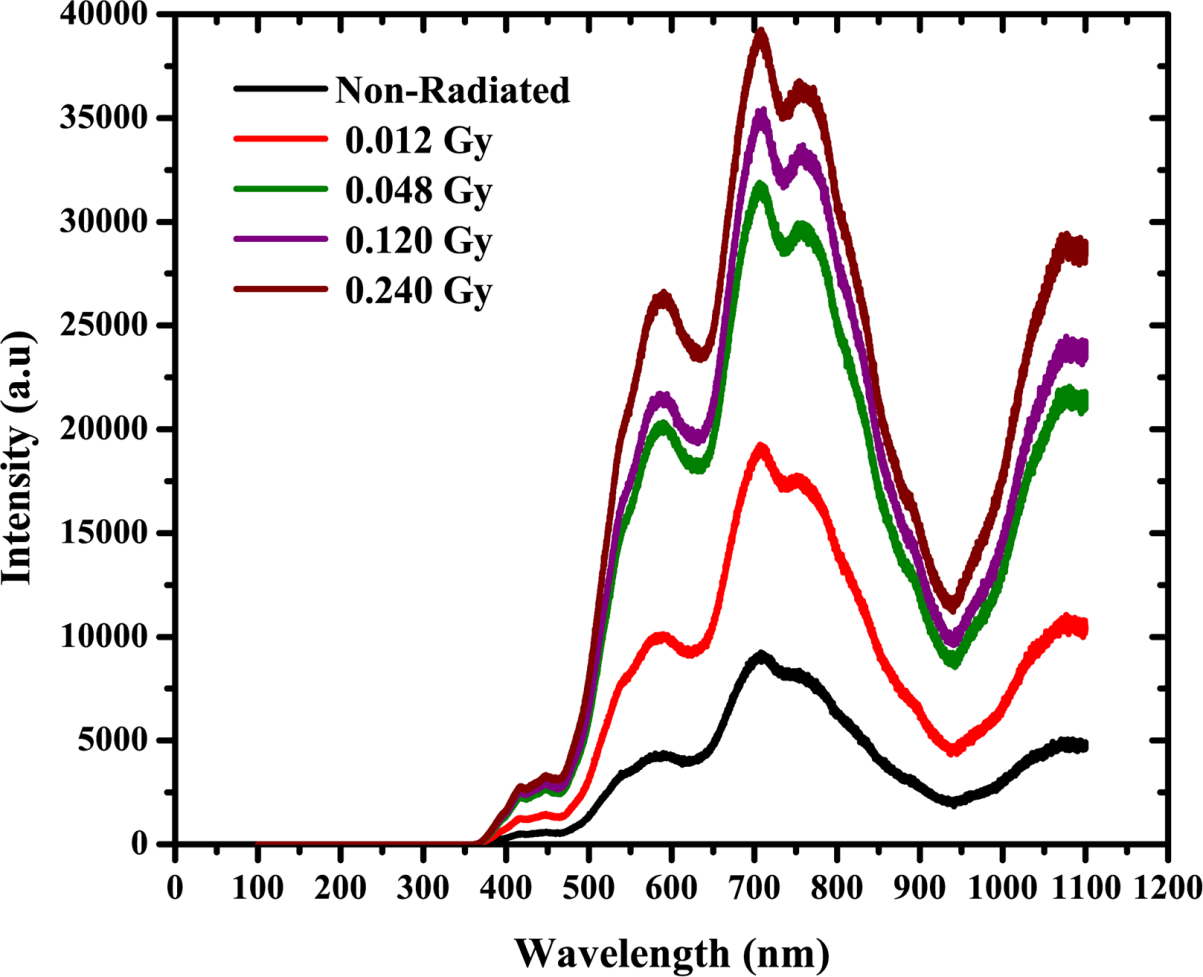
Photoluminescence spectra of DNA irradiated by alpha particles.

Generally excitation and emission are considered significant processes in phosphors luminescence. Different types of energy could excite the phosphors, such as photon (often ultra-violet) that acts to excite the PL, an electric voltage to excite electroluminescence (EL) or chemiluminescence excited by the energy of a chemical reaction, and so on. The process of emission is a release of energy in the form of photon [53]. Therefore, the results demonstrated in this current work may suggest utilization as light emitting structures based on DNA diodes show Fig 11.

## Conclusions

In this study, we fabricated surface-type ITO/DNA/Au Schottky barrier diodes and carried-out I-V measurements at room temperature. The electronic parameters such as the ideality factor, height barrier, series resistance and Richardson constant of the diode were also measured. In summary, we studied I–V characteristics of ITO/DNA/Au junctions exposed to different dosage (0–0.24 Gy). The metal-semiconductor-metal devices fabricated in this work demonstrated non-ideal I–V behavior. Barrier height determined from the I–V measurements were calculated at 0.7284 eV for non-radiation, increasing to about 0.7883 eV in 0.036 Gy of radiation, showing an increase for all doses. The radiation-induced hypersensitivity phenomena of the DNA used can be attributed to the changes observed in the ideality factors and series resistance values. It is therefore possible to utilize such fluctuations in Au/DNA/ITO junctions to enable rapid and accurate sensing or detection of alpha particles, also can be utilizing these devices as light emitting.
